# Electrocardiogram Features of Left Ventricular Excessive Trabeculation with Preserved Cardiac Function in Light of Cardiac Magnetic Resonance and Genetics

**DOI:** 10.3390/jcm13195906

**Published:** 2024-10-03

**Authors:** Kristóf Attila Farkas-Sütő, Kinga Grebur, Balázs Mester, Flóra Klára Gyulánczi, Csaba Bödör, Hajnalka Vágó, Béla Merkely, Andrea Szűcs

**Affiliations:** 1 Heart and Vascular Center, Semmelweis University, Városmajor utca 68., 1122 Budapest, Hungary; farkas.kristof@stud.semmelweis.hu (K.A.F.-S.);; 2 Department of Pathology and Experimental Cancer Research, Semmelweis University, Üllői út 26., 1085 Budapest, Hungary

**Keywords:** left ventricular excessive trabeculation, noncomapction, ECG, cardiac magnetic resonance imaging, cardiogenetics

## Abstract

**Background and Objectives**: Although left ventricular excessive trabeculation (LVET) can cause heart failure, arrhythmia and thromboembolism, limited literature is available on the ECG characteristics of primary LVET with preserved left ventricular function (EF). We aimed to compare the ECG characteristics and cardiac MR (CMR) parameters of LVET individuals with preserved left ventricular EF to a control (C) group, to identify sex-specific differences, and to compare the genetic subgroups of LVET with each other and with a C population. **Methods**: In our study, we selected 69 LVET individuals (EF > 50%) without any comorbidities and compared them to 69 sex- and age-matched control subjects (42% females in both groups, *p* = 1.000; mean age LVET-vs-C: 38 ± 14 vs. 38 ± 14 years *p* = 0.814). We analyzed the pattern and notable parameters of the 12-lead ECG recordings. We determined the volumetric and functional parameters, as well as the muscle mass values of the left and right ventricles (LV, RV) based on the CMR recordings. Based on the genotype, three subgroups were established: pathogenic, variant of uncertain significance and benign. **Results**: In the LVET group, we found normal but elevated volumetric and muscle mass values and a decreased LV_EF, wider QRS, prolonged QTc, higher RV Sokolow index values and lower T wave amplitude compared to the C. When comparing MR and ECG parameters between genetic subgroups, only the QTc showed a significant difference. Over one-third of the LVET population had arrhythmic episodes and a positive family history. **Conclusions**: The subclinical morphological and ECG changes and the clinical background of the LVET group indicate the need for follow-up of this population, even with preserved EF.

## 1. Introduction

The excessive apical trabeculation of the left ventricle (LVET) is known to have a broad spectrum of clinical manifestations; individuals with this morphology may experience heart failure with reduced LV ejection fraction (EF) and arrhythmic or thromboembolic events [1,2]. On the other end of this spectrum, a considerable part of the population lives with LVET morphology and preserved LVEF without experiencing any symptoms throughout their life [2,3]. However, differentiating between a healthy individual with an increased amount of trabeculae and asymptomatic, primary LVET individuals that are likely to develop complications can be challenging. As detailed in the current cardiomyopathy guideline published by the European Society of Cardiology, proper risk assessment is essential in order to avoid over-diagnosing and determine whether a LVET patient needs regular follow-ups or medical treatment [4].

As part of the algorithm, cardiac magnetic resonance imaging (CMR) plays a substantial role not only in the diagnosis of the condition but in the risk stratification process as well since changes in the volumetric and functional parameters are considered to be alarming red flags. Genetic testing, becoming more and more accessible, is also aiding clinicians in this decision-making process, although it is worth mentioning that individuals with pathogenic LVET mutations may also be asymptomatic with preserved LVEF [2,5,6,7].

Finally, an important part of this risk assessment is the analysis of electrocardiogram (ECG) and Holter examinations, as well as the investigation of the personal and family medical history of arrhythmic events [5,6]. Compared to the noncompact cardiomyopathy (NCMP) population with reduced LVEF, where branch blocks, malignant ventricular arrhythmias and sudden cardiac death (SCD) are known to be of higher prevalence, the literature data on the ECG characteristics of LVET individuals with preserved LVEF is very limited [8,9,10,11]. Furthermore, the intersex difference of ECG parameters is an underexplored area, as well.

Thus, our aim was to compare the ECG characteristics and CMR parameters of LVET individuals with preserved LVEF to a control (C) group, to identify sex-specific differences, and to compare the genetic subgroups of LVET with each other and with a C population.

## 2. Materials and Methods

### 2.1. Study Population

Our team maintains a detailed database of individuals with LVET morphology confirmed by CMR. In our retrospective study, we enrolled 69 (29 female, mean age: 37.7 ± 13.9 years) LVET individuals from this database (Table 1). Inclusion criteria were fulfilling both the Petersen and the Jacquier criteria for noncompaction normal LV and RV end-diastolic volumes and preserved ejection fraction (LVEF > 50%) [12,13,14]. We have excluded patients with reduced LVEF (LVEF < 50%); congenital, ischemic or significant valvular heart diseases; hypertension; other cardiomyopathies; sports activity exceeding 6 h/week; relevant extracardiac comorbidities, as well as conditions that can cause secondary hypetrabeculation, or patients whose CMR images were not usable due to technical reasons (i.e., artefacts, contrast administration before the short axis sequence) [15]. We also excluded subjects who were under medications known to elongate the QT. Based on the genetic testing results, the LVET group was divided into three subgroups detailed in the Section 2.4.

Regarding the control (C) group (69 individuals, 29 female, mean age: 38.2 ± 13.5 years), we carefully reviewed the anamnestic data of the population and included only individuals without comorbidities (e.g., hypertension, ischemic heart disease, cardiomyopathy, congenital anomalies, extracardiac disorders, etc.) with no abnormalities on the cardiac MRI and <6 h/week of sports activity (Table 1).

All procedures performed in this study were in accordance with the 1964 Helsinki Declaration and its later amendments or comparable ethical standards. Ethical approval was obtained from the Medical Research Council of Hungary and issued by the National Institute of Pharmacy and Nutrition. All participants (or their legal guardians) provided informed written consent. The patient data, including the ECG recordings, CMR examinations, genetic results and medical records, were accessed during the data collection from 2 October 2023 to 31 January 2024. All data that could identify individuals in the study were only accessed by the lead investigator (A.S.).

### 2.2. ECG Registration and Analysis

All subjects had 12-lead ECGs taken with a 25 mm/sec paper speed using Heart Screen 112 C-1 (Innomed Medical, Budapest, Hungary) and BTL 08-LC (BTL Industries, Stevenage, UK) ECG machines. All measurements were made manually using an ECG ruler by two independent readers, K.G. and K.A.F-S. To evaluate interobserver variability, 35 ECG registrations were assessed by both examiners, demonstrating excellent agreement across all parameters (Table S1). We calculated the average of five representative beats for each parameter, avoiding extrasystolic beats. Of the studied parameters, we measured the heart rate, the duration of the P and T waves, the PQ and QT interval and the amplitude of the P and T wave in lead II. QRS was measured in the lead, where it appeared the widest; in most cases, it was in either V3 or V4. The corrected QT (QTc) was calculated via the Bazett method. The left and right ventricular (RV) Sokolow–Lyon index (SI) was calculated by using leads V1 and V6. The ECGs were also analyzed for any signs suggestive of arrhythmia or intraventricular conduction disorder. We used the recommendations published by the American Hearts Association, American College of Cardiology Foundation and Heart Rhythm Society for reference values [16,17,18].

### 2.3. CMR Image Acquisition and Analysis

The CMR images were acquired using Magnetom Aera (Siemens Healthineers, Erlangen, Germany) and Achieva (Philips Medical System, Eindhoven, The Netherlands) 1,5T MR imaging machines. Conventional four-, three- and two-chamber long-axis cine loops, as well as short-axis (SA) cine images covering the whole LV and RV were recorded in breath-pause using retrospectively gated balanced steady-state free precession sequences. The slice thickness was 8 mm without an interslice gap, and the field of view was 350 mm on average, adapted to the body size. After the SA cine images were acquired, either gadobutrol (Gadovist, Bayer-Schering, 0.16 mL/kg) or gadobenate dimenglumine (MultiHance, Bracco, 0.25 mL/kg) was injected intravenously. Contrast agent was administered only to patients in the LVET group and only when it held added diagnostic value. The administration was performed after the short-axis cine loops to achieve the best analytic accuracy during post-processing. Late gadolinium enhancement was evaluated in each LVET subject. Overall, LGE was detected in 5 individuals showing a subepicardial or mid-myocardial pattern; however, due to the low number of cases, we performed no further investigation in this area.

The image analysis was performed using the Medis Suite software (Medis Suite QMass, version 4.0, Medis Medical Imaging Systems, Leiden, The Netherlands).

**Figure 1:**
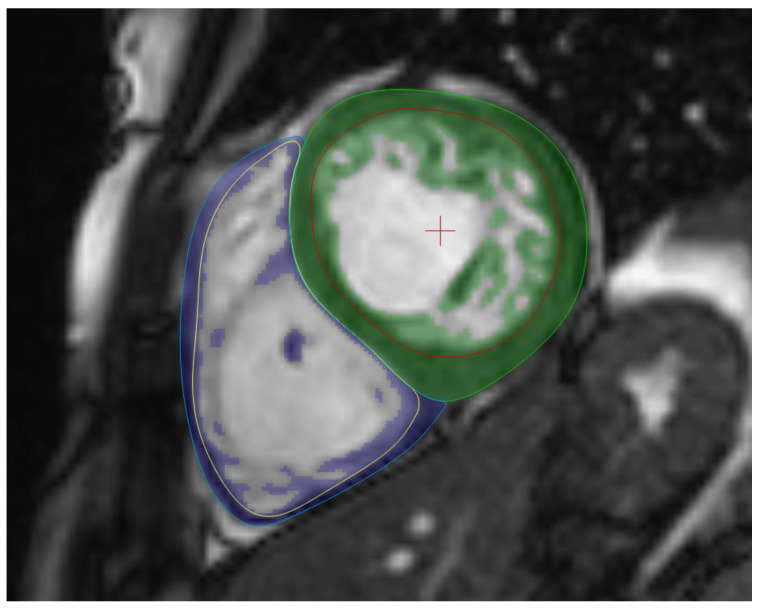
Threshold-based postprocessing method. The short-axis CMR image of an LVET individual with the threshold-based postprocessing method. The green highlight shows the LV, and the blue highlight shows the RV myocardial mass. The MassK algorithm analyzes each voxel and classifies them as either myocardial tissue or blood tissue; therefore, the highlighted myocardial tissue within the endocardial (red in the LV and yellow in the RV) border represents the TPM. Abbreviations: CMR, cardiac magnetic resonance imaging; LV, left ventricle; LVET, left ventricular excessive trabeculation; RV, right ventricle, TPM, trabeculated and papillary muscle mass.

During post-processing, the endocardial and epicardial tracings were made on the SA images in both the end-diastolic and end-systolic frames. We used a semi-automatic contouring technique with manual correction to fit the endo- and epicardial tracings to the inner and outer borders of the compact myocardium, respectively. The adjustments were made by two experienced readers: A.S., 14 years of experience, and Ba.M., with 4 years of experience. To evaluate interobserver variability, 20 CMR examinations were assessed by both examiners, demonstrating excellent agreement across all parameters (Table S1). The MassK algorithm was used, which is a threshold-based technique that analyzes each voxel and classifies them as either myocardial tissue or blood tissue (Figure 1). Therefore, the total myocardial mass (TM) represents the myocardial tissue within the epicardial border; while the trabeculated and papillary muscle mass (TPM) represents the myocardial tissue within the endocardial border. Both parameters were calculated by using the end-diastolic frame. Besides the muscle mass values, the software calculates the LV and RV end-diastolic, end-systolic and stroke volumes (EDV, ESV, SV) and the ejection fraction (EF). All volumetric and myocardial mass parameters were indexed to the body surface area (i). For reference values, we used the normal ranges established by Alfakih [19].

### 2.4. Genetic Testing

Genetic testing was performed on 55 symptomatic LVET subjects by using peripheral blood samples. For the analyses, we applied the next-generation sequencing method by using the TrueSight Cardio Sequencing kit (Illumina, CA, USA), which contained 174 genes previously associated with cardiovascular diseases.

During the sequencing reaction, paired-end reads of 150 nucleotides in length were synthesized. The fastq files converted from optical information were quality-controlled by using FastQC (v0.11.9) and MultiQC (v1.9) software. The obtained genetic information was compared to the GRCh37.p13 assembly version of reference genome hg19 with BWA software (v0.7.12). The Broad Institute GATK software (v4.1.7.0) was used to detect mutations, which was followed by manual variant screening.

The ClinGen and OMIM databases were used to identify the genes associated with CMP, and the genetic variants were assessed by using Franklin, ClinVar and VarSome databases following the recommendations of the American College of Medical Genetics and Genomics (ACMG) [20]. All variants were classified as either pathogenic (P), likely pathogenic (LP), variant of uncertain significance (VUS), likely benign and benign (B) based on clinical relevance (Tables S2 and S3).

In accordance with the ACMG guidelines, we divided the LVET subjects into three subgroups: the P group consisted of individuals with either P or LP mutations, the VUS group consisted of subjects with VUS and the B group consisted of individuals without any of these mutations (Table 1).

### 2.5. Clinical Background

In order to collect clinical and demographic data, LVET subjects filled out an anamnestic questionnaire containing both open-ended and closed questions. It included detailed questions about family and personal medical history, including arrhythmias, sudden cardiac death (SCD), implanted cardiac devices, structural or hereditary cardiac diseases, myocardial infarction, thromboembolic events, syncope, dizziness, chest pain and palpitation. The questionnaire also covered comorbidities, such as diabetes mellitus, kidney, hepatic, gastrointestinal, pulmonary, neurologic, endocrine, oncologic, rheumatic, autoimmune, and psychiatric diseases, metabolic syndromes and medications and sports activity. Furthermore, additional information was gathered by using available electronic medical records. When reviewing the medical history, we defined documented arrhythmia as conditions either diagnosed by Holter or ECG or those requiring treatment.

### 2.6. Statistical Analysis

All continuous parameters are reported as the mean ± standard deviation, and all discrete parameters are reported as counts or percentages. At first, we compared the CMR and ECG parameters between the LVET and the C groups. After that, we compared the CMR and ECG parameters of males and females in both the LVET and C groups separately. Then, we opposed the ECG parameters between the LVET and C subjects in both sexes separately. The Kolmogorov–Smirnov test was used to assess the normal distribution of continuous parameters, and Levene’s test was used to assess homogeneity. For the comparison of means between the two groups, we used independent-sample *t*-tests and Mann–Whitney U-tests dependent on the distribution of the dataset. To assess the difference between the genetic subgroups, for normally distributed parameters, we used the one-way analysis of variance (ANOVA) with Tukey’s post hoc test or the Welch test with the Games–Howel post hoc test dependent on the equality of variances between the subgroups. In the case of the non-normally distributed parameters, we used the Kurskal–Wallis test with Dunn’s post hoc test. We used Pearson’s correlation coefficient to assess the connection between the ECG and CMR parameters. To measure the interobserver variability, we used the intraclass correlation coefficient (ICC); values above 0.75 were considered to be excellent agreement. A *p*-value below 0.05 was considered statistically significant. IBM SPSS Statistics (Version 28.0, Armonk, NY, USA) was used for the statistical analyses.

## 3. Results

In this section we will present our results firstly by phenotype then by genotype and lastly we will describe the clinical characteristics of the LVET population.

### 3.1. Phenotype-Based Comparisons

#### 3.1.1. Comparison of CMR Parameters

When comparing the CMR results between the LVET population and the C group, we found all LV volumes, the RV_EDVi and RV_ESVi, as well as the LV_TMi, LV_TPMi and RV_TPMi, to be significantly increased and the LV_EF to be significantly decreased in the LVET group (Table 2).

The intersex comparison in the LVET population showed that the males had higher volumes and mass values in both LV and RV. In the C population, we only found significant intersex differences in the TPMi and TMi parameters (Table 2).

When examining the relationship between the volumetric and mass values of the LVET population, we found a good correlation between these CMR parameters (Table S4).

#### 3.1.2. Comparison of ECG Parameters

Regarding the comparison of ECG parameters between the LVET and C groups, the duration of the P wave, the QRS, the QT and the QTc were significantly longer, the P and T wave amplitudes were significantly lower and the RV_SI was significantly higher (Table 2).

When examining the relationship between ECG and CMR parameters, we found that the QRS duration and the LV_SI and RV_SI showed a significant but overall moderate correlation with the volumetric and the muscle mass CMR parameters (Table 3).

Regarding the male population, in subjects with the LVET morphology, the P wave, the QRS, the QT and the QTc durations were significantly longer, the P and T wave amplitudes were significantly lower and the RV_SI was significantly higher compared to C. However, in the female population, only the QTc duration and the T wave amplitude showed significant differences between the LVET and C groups, which were similar to those of men (Table 2).

**Figure 2:**
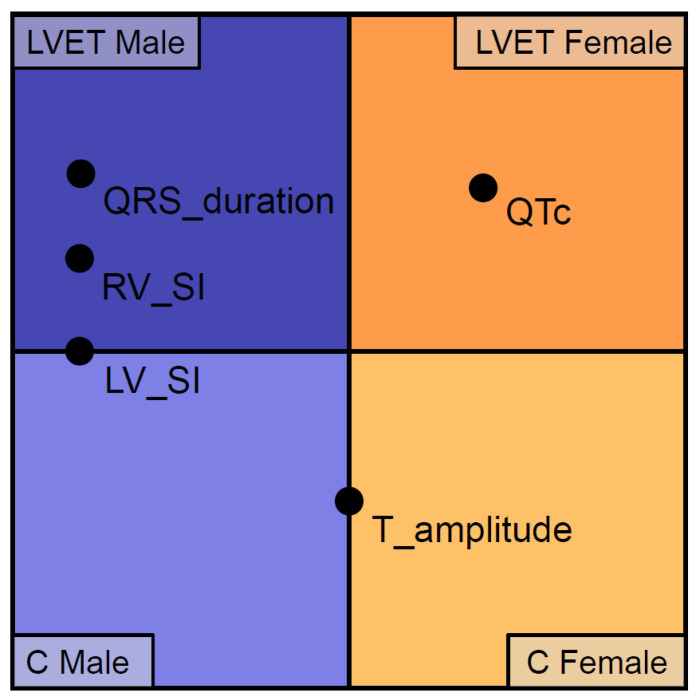
ECG characteristics in the aspect of morphology and sex. QRS_duration: LVET male characteristic; LV_SI: male characteristic, LVET independent; RV_SI: LVET male characteristic; QTc: female characteristic, prolonged in LVET; T_amplitude: characteristic to healthy controls, sex independent. Abbreviations: LVET, left ventricular excessive trabeculation; C, control population; LV, left ventricle; RV, right ventricle; SI, Sokolow–Lyon index.

Then, we examined the differences between sexes regarding the ECG parameters within each population. In the LVET population, males had significantly higher P wave and QRS duration, LV_SI and RV_SI values and significantly decreased QTc duration compared to females. We found similar results in the C group, except for the QRS duration, which was comparable between the sexes (Figure 2, Table 2).

### 3.2. Genetic Aspects of the Measured Parameters

**Figure 3:**
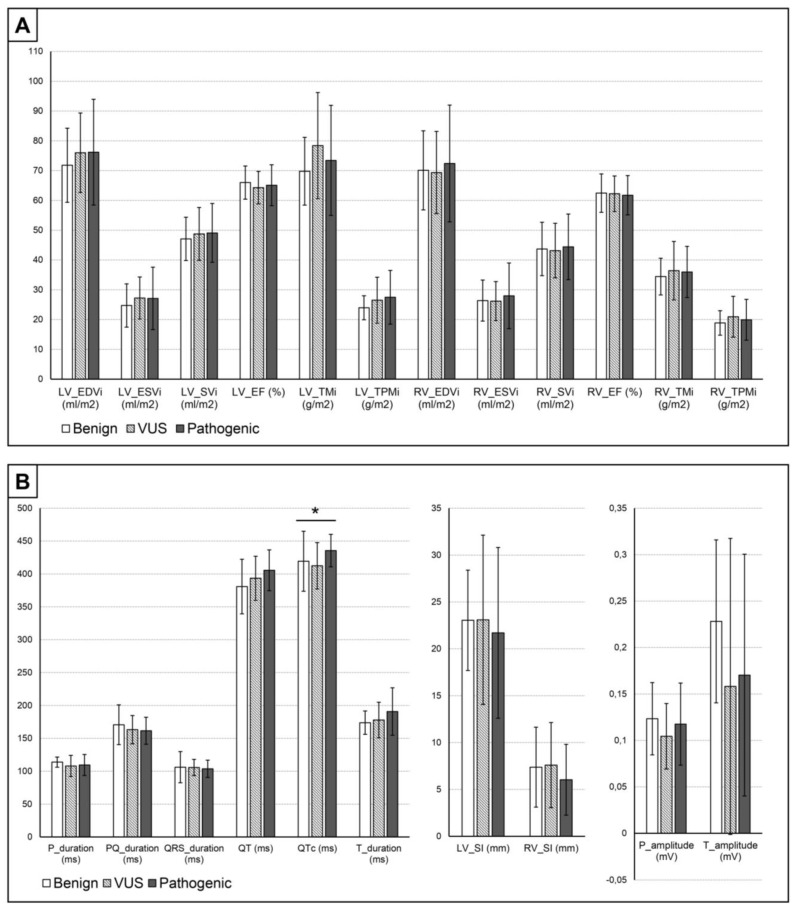
Comparisons of the (**A**) CMR and (**B**) ECG parameters between the genetic subgroups. Abbreviations: * significant at the *p* < 0.05 level; EDV, end-diastolic volume; EF, ejection fraction; ESV, end-systolic volume; i, indexed to body surface area; LV, left ventricle; LVET, left ventricular excessive trabeculation; QTc, corrected QT interval; RV, right ventricle; SI, Sokolow–Lyon index; SV, stroke volume; TM, total muscle mass; TPM, trabeculated and papillary muscle mass; VUS, variant of unknown significance.

When comparing the three genetic subgroups with each other, none of the CMR parameters showed significant differences. However, among the ECG parameters, the QTc duration was significantly different between the VUS and P subgroups (Figure 3).

We also compared the ECG parameters of each genetic subgroup with their respective control group. Regarding the B subgroup, only the P wave duration showed a significant difference compared to controls. The VUS population showed significantly higher QRS and QT durations, lower P and T wave amplitudes and a higher RV_SI than the C group. The P subgroup had a significantly longer P, QT and QTc duration, as well as lower T amplitudes (Table 4).

Regarding the QRS duration and RV_SI, their correlation with the CMR parameters became stronger when examining the P subgroup separately (Table S5).

### 3.3. ECG Characteristics and Clinical Parameters of the LVET Group

After reviewing the ECG pattern of the LVET subjects, it is notable that a third of the population had T wave inversion in at least two corresponding leads, and almost two-thirds had QRS anomalies suggestive of depolarization disorder; out of those, 40% had an incomplete right bundle branch block (RBBB). See details in Table 5.

Regarding the clinical characteristics of the LVET group, arrhythmia had been documented in 39% of the population, and five people had experienced syncope before (vasovagal syncope episodes have been excluded) (Table 5). One third of the LVET individuals had a positive family history, which we defined as having a blood relative who had been either diagnosed with familial cardiomyopathy, experienced SCD or had an implantable cardioverter-defibrillator (ICD) device or malignant ventricular tachycardia (Table 5). It is worth mentioning that this ratio almost doubled if we examined the P group separately.

## 4. Discussion

Since LVET has a broad spectrum of clinical manifestations ranging from healthy individuals to severe heart failure, risk assessment—including the analysis of CMR, ECG examinations and genetic results—is essential to determine the optimal patient management, even in symptomless individuals. Thus, in our retrospective study, we analyzed these above-mentioned parameters in an LVET population with preserved LVEF.

### 4.1. Phenotype-Based Comparisons

Firstly, we compared the CMR parameters of the LVET and C groups. The LVET population had normal but elevated LV and RV volumes and higher LV and RV TPMi values but decreased LV_EF compared to the normal population, which coincides with other publications [21,22]. Interestingly, in contrast to our and other CMR studies, Horváth et al. found comparable RV volumes and decreased RV_EF with 3D echocardiography when compared to controls [23,24].

Regarding the intersex comparisons of the LVET population, the CMR results of Kiss et al. are in line with ours; namely, the LV volumetric and muscle mass parameters are elevated in males compared to females [21]. In the C population, similarly to Gregor et al. the LV volumetric results were comparable between sexes in the same age group; however, Kiss et al. found significant differences when studying a larger cohort [21,25,26]. Regarding the RV, we found no significant intersex difference in the volumes of the C group, which could be related to the lower number of subjects [26,27]. However, more notable is the significant difference between sexes in the volumetric and muscle mass values in the LVET population.

Secondly, we compared the ECG parameters between LVET and C groups in the whole population and also in each sex separately. Then, we examined the intersex differences in both the LVET and the C populations.

#### 4.1.1. Horizontal and Vertical Characteristics of the Depolarization

The QRS duration was in the normal range but significantly longer in the LVET population compared to C and correlated with the LV and RV volumes and mass values; however, this trait was only characteristic of LVET men. On the contrary, the QRS duration did not differ significantly between the sexes in the C group.

The positive correlation between the QRS duration and the TPM might be explained by the fact that the more excessive the trabeculation is the longer pathway the stimulus has to pass through during systole and it is not known how far the Purkinje fibers extend into the trabeculae.

Domain et al. also compared the QRS duration of decreased LVEF NCMP patients to a healthy population and found it to be longer and correlated with the LV muscle mass, which is comparable to our results [28]. Ergul et al. also found similar differences regarding the QRS duration of LVET children population with mixed LVEF [29]. Hnatkova et al. examined the QRS characteristics of a healthy cohort, and contrary to our results, they found its duration to be longer in men, which may be due to the significantly larger number of subjects investigated and different measurement techniques [30]. However, we have not found detailed literature data on the ECG characteristics of LVET subjects with preserved LVEF.

Examining the vertical parameters, while the LV-SI does not seem to be LVET-dependent, the RV_SI value was significantly higher in LVET men compared to C men; however, we found both SI values to be higher in men than women, regardless of the left ventricular phenotype. These findings are coherent with both the intersex CMR differences in TPMi and TMi values of the LVET population and the available literature data [31]. Furthermore, similarly to the QRS duration, the SI values showed significant correlations with the LV and RV volumes and mass values of the LVET population. These findings could be corresponding to the ECG differences of the characteristic morphological features of the population, namely the normal but elevated volumes and mass parameters.

Regarding the P wave, we found it to be longer in duration but lower in amplitude in the LVET population compared to C; however, the literature data regarding the atrial characteristics of the population are limited, and the significance of this difference is yet to be studied [29,32].

#### 4.1.2. Horizontal and Vertical Characteristics of the Repolarization

The QTc duration was within the normal range but significantly longer in the LVET population, and we found it to be significantly longer in females compared to males. On the one hand, the prolonged QTc is characteristic of LVET, as proven by previous studies; however, they investigated a population with mixed LVEF [9,29,32]. This difference could be the reason behind our QTc durations still being in the normal range, although the significant difference compared to C may suggest subclinical changes even while the LVEF is preserved.

On the other hand, women are known to have longer QTc durations than men due to the different levels of the sexual steroid hormones, which coincide with our results in both the LVET and C groups [33,34].

Although the T wave duration was comparable between phenotypes, the T wave amplitude was significantly lower in LVET subjects compared to the C group regardless of their sex; however, there was no intersex difference in either population. Ezaki et al. studied the intersex differences of the T wave amplitude in adolescence in a large cohort and found men to have significantly higher values than women; however, they used different measurement techniques [35].

### 4.2. Phenotype- and Genotype-Based Comparison

When comparing the genetic subgroups with each other, we found no significant difference in the CMR parameters, and only the QTc differed significantly out of the ECG parameters. This could be caused both by the low number of subjects in the B and P LVET subgroups compared to the VUS subgroup and the different ratios of sexes and mean age between the subgroups. Van Waning et al. and Grebur et al. had similar results when comparing the CMR parameters of LVET subjects based on genotype [7,36]. Miszalski-Jamka et al. found the LVEF of those carrying P mutations to be significantly lower, although it is worth mentioning that the study populations had reduced systolic LV function [37].

However, when comparing each genetic subgroup to their age- and sex-matched C groups, we found that the depolarization and repolarization phases differed significantly in both VUS and P subgroups despite the low number of subjects. Interestingly, the B subgroup was comparable to controls in that regard. Furthermore, when examining the relationship between the QRS duration and RV_SI values and CMR parameters, the correlation was much stronger in the P group separately than in the whole LVET group. Regarding the literature data, we could not find any publications on the genotype-based comparison of the ECG parameters between the LVET and C populations.

### 4.3. ECG Findings and Clinical Aspects of the LVET Group

Finally, the NCMP population is known to have a higher rate of T wave anomalies, as well as a fragmented QRS morphology originating from depolarization disorders, which is associated with poor outcomes; however, they have only been studied on a population with reduced LVEF [11,29,32,38,39]. In our study, we showed that the T wave inversion and fragmented QRS morphology, to some extent, also appear in the LVET population with preserved LVEF, although its prognostic significance in this context is yet to be studied.

Regarding the clinical characteristics, we observed a high prevalence of arrhythmic events and positive family history among the LVET population, which are considered to be alarming symptoms according to the risk-stratification protocol [5,6]. Grebur et al. found that the LVET individuals carrying P mutations had a higher number of these so-called “red flags”, underlining the connections between genotype and phenotype, as well as the importance of genetic testing in LVET individuals with preserved LVEF [7].

Overall, we found ECG depolarization and repolarization abnormalities characteristic to LVET, some of which seem to be sex-dependent and associated with genetic background. The literature data on the ECG characteristics of LVET with preserved LVEF are very limited, especially in the context of the above-mentioned aspects. Our findings raise the possibility of a subclinical effect of excessive trabeculation on the electrophysiology of the LVET population, especially with the relatively high prevalence of depolarization disorders, T wave inversions and arrhythmic events in the population. However, further research is needed to determine the prognostic value of these abnormalities.

### 4.4. Limitations

Our study’s main limitation is the relatively small number of subjects, especially when examining the different genetic subgroups separately. We are planning on expanding our LVET repository with more genetic data in the future. Secondly, the genetic testing kit available to us is only sensitive to single-nucleotide polymorphism mutations but not to copy number variations. Finally, since our study was conducted retrospectively and most of the ECGs were not stored digitally, it was impossible for us to use more modern, software-based means of measurement.

## 5. Conclusions

We examined the ECG and CMR parameters of an LVET group with preserved LVEF, compared them to healthy controls and investigated the sex-specific characteristics of the population and their relationship with genetics.

The LVET population had normal but elevated LV and RV volumes and higher TPMi values but decreased LV_EF compared to the normal population, which was more pronounced in males compared to females. The QRS duration and the RV_SI were significantly increased in LVET males compared to both females and C, and they correlated with the LV and RV volumes and muscle mass values. The QTc duration was significantly longer in the LVET population, and—similarly to the healthy population—we found it to be significantly longer in females compared to males. The T wave amplitude was significantly lower in LVET subjects compared to the C group without an intersex difference.

Of the measured CMR and ECG parameters, there were no significant differences between the genetic subgroups, and while we found the B subgroup to be comparable to C, the VUS and P subgroups differed significantly from healthy subjects in several parameters.

The subclinical changes in the ECG and CMR parameters of the LVET population, in conjunction with the high prevalence of arrhythmic events and positive family history, highlight the need for regular follow-ups even with preserved LVEF.

## Figures and Tables

**Table 1:** Demographic data of the (**A**) LVET and control populations and (**B**) genetic subgroups.

A	LVET			Control			LVET Total vs. C Total	LVET Male vs. C Male	LVET Female vs. C Female
	Total Population	Male	Female	Total Population	Male	Female	*p*	*p*	*p*
Number of subjects (%)	69 (100.0)	40 (58.0)	29 (42.0)	69 (100.0)	40 (58.0)	29 (42.0)	1.000	1.000	1.000
Age (Years)	37.7 ± 13.9	37.9 ± 14.5	37.3 ± 13.5	38.2 ± 13.5	38.8 ± 13.6	37.3 ± 13.4	0.814	0.776	0.985
BMI (kg/m^2^)	1.9 ± 0.2	2.1 ± 0.2	1.7 ± 0.1	1.9 ± 0.2	2.0 ± 0.1	1.7 ± 0.1	0.255	0.228	0.307
BSA (m^2^)	25.4 ± 4.4	26.4 ± 3.9	24.0 ± 4.7	24.4 ± 4.2	25.9 ± 3.8	22.4 ± 4.1	0.190	0.563	0.184
**B**	**Genetic subgroups**								
	**Benign**	**VUS**	**Pathogenic**	***p***					
Number of subjects (Female %)	13 (30.1)	27 (37.0)	15 (53.3)	0.551					
Age (Years)	40.6 ± 4.1	39.4 ± 2.4	37.1 ± 4.2	0.789					
BMI (kg/m^2^)	2.02 ± 0.27	1.98 ± 0.23	1.84 ± 0.20	0.094					
BSA (m^2^)	26.21 ± 4.87	25.94 ± 4.10	23.37 ± 3.12	0.106					

Abbreviations: C, control; LVET, left ventricular excessive trabeculation; VUS, variant of unknown significance.

**Table 2:** CMR and ECG parameters of the studied populations.

	LVET			Control			LVET Total vs. C Total	LVET Male vs. LVET Female	C Male vs. C Female	LVET Male vs. C Male	LVET Female vs. C Female
	Total Population	Male	Female	Total Population	Male	Female	*p*	*p*	*p*	*p*	*p*
Number of subjects	69	40	29	69	40	29					
LV_EDVi (ml/m^2^)	76.4 ± 14.2	81.6 ± 13.5	69 ± 11.9	66.9 ± 10.1	68.6 ± 11.0	64.4 ± 8.4	<0.001 *	<0.001 *	0.094	-	-
LV_ESVi (ml/m^2^)	27.1 ± 8.0	29.4 ± 7.9	23.7 ± 6.9	21.2 ± 5.4	21.6 ± 6.0	20.6 ± 4.4	<0.001 *	0.003 *	0.471	-	-
LV_SVi (ml/m^2^)	49.4 ± 8.8	52.2 ± 8.4	45.3 ± 7.8	45.7 ± 7.4	47.0 ± 8.3	43.8 ± 5.6	<0.001 *	0.001 *	0.061	-	-
LV_EF (%)	64.9 ± 5.9	64.2 ± 5.7	66.0 ± 6.3	68.4 ± 5.6	68.6 ± 6.3	68.1 ± 4.3	0.009	0.244	0.725	-	-
LV_TMi (g/m^2^)	75.2 ± 16.8	85.7 ± 12.4	60.1 ± 8.9	66.7 ± 11.2	72.4 ± 10.1	58.5 ± 6.9	<0.001 *	<0.001 *	<0.001 *	-	-
LV_TPMi (g/m^2^)	26.1 ± 7.4	29.4 ± 7.0	21.3 ± 4.9	20.3 ± 4.2	21.8 ± 4.1	18.0 ± 3.2	<0.001 *	<0.001 *	<0.001 *	-	-
RV_EDVi (ml/m^2^)	71.6 ± 14.4	77.9 ± 13.2	62.5 ± 10.9	65.8 ± 12.6	67.9 ± 13.4	62.8 ± 11.0	0.014 *	<0.001 *	0.1	-	-
RV_ESVi (ml/m^2^)	27.0 ± 6.7	29.9 ± 5.9	22.9 ± 5.4	24.1 ± 6.1	25.2 ± 6.5	22.6 ± 5.3	0.01 *	<0.001 *	0.089	-	-
RV_SVi (ml/m^2^)	44.3 ± 8.9	47.5 ± 7.6	39.6 ± 8.8	41.6 ± 8.7	42.7 ± 9.5	40.2 ± 7.2	0.083	<0.001 *	0.238	-	-
RV_EF (%)	62.0 ± 5.9	61.2 ± 4.6	63.2 ± 7.3	63.3 ± 5.5	62.8 ± 5.9	64.1 ± 4.9	0.183	0.207	0.342	-	-
RV_TMi (g/m^2^)	36.8 ± 9.7	42.2 ± 8.8	29.4 ± 5.0	35.1 ± 7.1	38.4 ± 6.2	30.2 ± 5.6	0.479	<0.001 *	<0.001 *	-	-
RV_TPMi (g/m^2^)	20.7 ± 7.0	24.8 ± 5.8	14.8 ± 3.4	18.3 ± 4.6	20.1 ± 4.3	15.7 ± 3.6	0.022 *	<0.001 *	<0.001 *	-	-
P_duration (ms)	108.3 ± 15.8	111.7 ± 14.8	103.4 ± 16.2	101.5 ± 13.6	104.8 ± 13.3	96.8 ± 13.0	0.007 *	0.034	0.017 *	0.031 *	0.099
P_amplitude (mV)	0.11 ± 0.04	0.11 ± 0.04	0.12 ± 0.04	0.13 ± 0.04	0.13 ± 0.04	0.13 ± 0.05	0.018 *	0.085	0.91	0.005 *	0.581
PQ_duration (ms)	163.1 ± 25.6	166.4 ± 26.0	158.4 ± 24.8	156.8 ± 19.7	160.6 ± 17.9	151.5 ± 21.2	0.85	0.204	0.059	0.248	0.268
QRS_duration (ms)	105.7 ± 15.8	112.0 ± 15.1	96.6 ± 12.4	99.5 ± 11.4	100.1 ± 11.4	98.5 ± 11.5	0.01 *	<0.001 *	0.429	<0.001 *	0.581
LV_SI (mm)	22.4 ± 8.3	24.9 ± 8.8	18.9 ± 5.8	23.0 ± 6.9	24.4 ± 6.2	21.0 ± 7.5	0.653	0.001 *	0.046 *	0.759	0.23
RV_SI (mm)	7.2 ± 4.2	8.5 ± 4.1	5.3 ± 3.7	5.3 ± 2.6	5.9 ± 3.4	4.8 ± 2.1	0.012 *	<0.001 *	0.049 *	0.003 *	0.967
QT (ms)	395.5 ± 35.0	394.8 ± 36.3	396.4 ± 33.9	378.3 ± 26.7	370.3 ± 25.1	389.1 ± 26.3	0.002 *	0.848	0.004 *	<0.001 *	0.37
QTc (ms)	423.3 ± 36.6	413.0 ± 35.1	438.1 ± 34.1	407.3 ± 24.9	398.7 ± 25.3	418.7 ± 18.7	0.003 *	0.005 *	0.002 *	0.048 *	0.012 *
T_duration (ms)	180.9 ± 29.1	181.9 ± 34.1	179.5 ± 20.6	176.4 ± 19.3	175.0 ± 18.4	178.3 ± 20.6	0.288	0.726	0.487	0.267	0.831
T_amplitude (mV)	0.20 ± 0.14	0.20 ± 1.6	0.19 ± 0.12	0.27 ± 0.10	0.28 ± 0.11	0.26 ± 0.07	0.002 *	0.832	0.531	0.025 *	0.014 *

Abbreviations: * correlation is significant at the *p* < 0.05 level; C, control; EDV, end-diastolic volume; EF, ejection fraction; ESV, end-systolic volume; i, indexed to body surface area; LV, left ventricle; LVET, left ventricular excessive trabeculation; QTc, corrected QT interval; RV, right ventricle; SI, Sokolow–Lyon index; SV, stroke volume; TM, total muscle-mass; TPM, trabeculated and papillary muscle mass.

**Table 3:** The correlation of the CMR and ECG parameters in the LVET group.

LVET Group		LV_EDVi	LV_ESVi	LV_SVi	LV_EF	LV_TMi	LV_TPMi	RV_EDVi	RV_ESVi	RV_SVi	RV_EF	RV_TMi	RV_TPMi
P_duration (ms)	r	0.037	0.081	−0.014	−0.069	0.097	0.092	0.189	0.187	0.099	−0.152	0.215	0.193
	p	0.765	0.516	0.913	0.579	0.433	0.459	0.126	0.13	0.425	0.22	0.081	0.119
P_amplitude (mV)	r	−0.119	−0.119	−0.084	0.078	−0.353 **	−0.382 **	−0.135	−0.131	−0.137	−0.002	−0.151	−0.249 *
	p	0.338	0.336	0.5	0.53	0.003	0.001	0.275	0.292	0.268	0.987	0.223	0.042
PQ_duration (ms)	r	−0.006	0.015	−0.025	−0.01	0.085	0.045	0.086	0.137	0.026	−0.098	0.088	0.14
	p	0.96	0.902	0.844	0.936	0.492	0.715	0.487	0.268	0.835	0.429	0.477	0.258
QRS_duration (ms)	r	0.269 *	0.287 *	0.172	−0.214	0.521 **	0.451 **	0.425 **	0.379 **	0.341 **	−0.117	0.446 **	0.427 **
	p	0.028	0.018	0.165	0.083	<0.001	<0.001	<0.001	0.002	0.005	0.347	<0.001	<0.001
LV_SI (mm)	r	0.349 **	0.241 *	0.343 **	−0.065	0.409 **	0.303 *	0.198	0.145	0.271 *	0.08	0.353 **	0.353 **
	p	0.004	0.05	0.004	0.604	0.001	0.013	0.108	0.24	0.026	0.518	0.003	0.003
RV_SI (mm)	r	0.421 **	0.357 **	0.356 **	−0.142	0.413 **	0.294 *	0.359 **	0.290 *	0.329 **	−0.034	0.445 **	0.343 **
	p	<0.001	0.003	0.003	0.252	0.001	0.016	0.003	0.017	0.007	0.787	<0.001	0.004
QT (ms)	r	0.196	0.183	0.15	−0.129	0.196	0.096	0.232	0.167	0.251 *	0.047	0.272 *	0.161
	p	0.111	0.138	0.225	0.299	0.111	0.439	0.059	0.176	0.041	0.708	0.026	0.193
QTc (ms)	r	−0.114	−0.073	−0.117	−0.001	−0.227	−0.222	−0.111	−0.228	−0.021	0.183	−0.128	−0.257 *
	p	0.358	0.556	0.347	0.993	0.064	0.071	0.371	0.064	0.864	0.139	0.301	0.036
T_duration (ms)	r	0.113	0.022	0.161	0.052	−0.009	−0.134	0.144	0.17	0.127	−0.06	0.137	0.103
	p	0.365	0.857	0.194	0.679	0.941	0.281	0.244	0.17	0.307	0.632	0.269	0.409
T_amplitude (mV)	r	−0.005	−0.077	0.061	0.114	0.034	−0.094	0.145	0.198	0.059	−0.168	0.156	0.144
	p	0.966	0.534	0.623	0.359	0.786	0.451	0.243	0.107	0.638	0.174	0.208	0.245

Abbreviations: * correlation is significant at the *p* < 0.05 level; ** correlation is significant at the *p* < 0.01 level; EDV, end-diastolic volume; EF, ejection fraction; ESV, end-systolic volume; i, indexed to body surface area; LV, left ventricle; LVET, left ventricular excessive trabeculation; QTc, corrected QT interval; r, Correlation coefficient; RV, right ventricle; SI, Sokolow–Lyon index; SV, stroke volume; TM, total muscle mass; TPM, trabeculated and papillary muscle mass.

**Table 4:** ECG parameters of the genetic subgroups and their controls.

	Benign Subgroup			VUS Subgroup			Pathogenic Subgroup		
	LVET	Control	*p*	LVET	Control	*p*	LVET	Control	*p*
Number of subjects	13	13		27	27		15	15	
P_duration (ms)	113.8 ± 7.7	99.6 ± 16.9	<0.01 *	108.0 ± 16.1	106.0 ± 11.6	0.611	109.5 ± 16.0	99.1 ± 10.3	0.043 *
P_amplitude (mV)	0.12 ± 0.04	0.12 ± 0.05	0.4	0.10 ± 0.04	0.13 ± 0.04	0.009 *	0.12 ± 0.04	0.14 ± 0.04	0.196
PQ_duration (ms)	170.7 ± 30.3	161.5 ± 26.6	0.7	163.2 ± 21.5	160.6 ± 19.5	0.58	161.6 ± 20.6	150.7 ± 14.0	0.101
QRS_duration (ms)	106.2 ± 23.7	102.2 ± 11.4	0.3	105.7 ± 12.3	97.4 ± 10.4	0.01 *	103.7 ± 13.1	95.4 ± 13.4	0.097
LV_SI (mm)	23.0 ± 5.4	21.5 ± 6.8	0.7	23.1 ± 9.0	22.9 ± 7.1	0.947	21.7 ± 9.1	24.0 ± 7.6	0.452
RV_SI (mm)	7.4 ± 4.3	5.0 ± 2.8	0.8	7.6 ± 4.5	5.3 ± 2.3	0.047 *	6.0 ± 3.8	4.7 ± 2.5	0.308
QT (ms)	380.7 ± 41.5	381.1 ± 26.0	0.7	393.4 ± 33.5	375.3 ± 21.8	0.023 *	405.6 ± 31.0	374.8 ± 35.7	0.018 *
QTc (ms)	419.3 ± 45.6	415.3 ± 20.4	0.8	412.4 ± 35.2	399.9 ± 27.5	0.156	435.5 ± 24.7	406.3 ± 26.8	0.04 *
T_duration (ms)	173.8 ± 17.8	171.2 ± 21.0	0.4	177.9 ± 27.1	175.6 ± 22.0	0.738	190.8 ± 36.1	176.9 ± 15.9	0.184
T_amplitude (mV)	0.23 ± 0.09	0.27 ± 0.14	0.6	0.16 ± 0.16	0.26 ± 0.09	0.014 *	0.17 ± 0.13	0.27 ± 0.07	0.019 *

Abbreviations: * significant at the *p* < 0.05 level; LV, left ventricle; LVET, left ventricular excessive trabeculation; QTc, corrected QT interval; RV, right ventricle; SI, Sokolow–Lyon index; VUS, variant of unknown significance.

**Table 5:** Clinical parameters of the LVET group.

			Number of Subjects (%)
	Total population		69 (100)
ECG abnormalities	P pulmonale		1 (1.4)
	P mitrale		7 (10.4)
	Atrioventricular block I. degree		5 (7.2)
	Depolarization disorders		
		fQRS	45 (65.2)
		LBBB	2 (2.9)
		RBBB	2 (2.9)
		Incomplete RBBB	16 (23.2)
		Left anterior fascicular block	5 (7.2)
		Preexitation	1 (1.4)
	Repolarization abnormalities		
		ST segment depression	3 (4.3)
		J point elevation	2 (2.9)
		T wave inversion	24 (34.8)
		QTc prolongation	10 (14.5)
Clinical characteristics	Positive family history		26 (38.7)
	Documented arrhythmia		27 (39.1)
		Supraventricular arrhythmia	13 (18.8)
		AVNRT	3 (4.3)
		AVRT	0
		Atrial fibrillation	5 (7.3)
		Ventricular arrhythmia	24 (34.8)
		VES	20 (29.0)
		Ventricular tachycardia	6 (8.7)
		Bradycardia	8 (11.6)
		Sudden Cardiac Death	1 (1.5)
	Thromboembolic events		2 (2.9)

Abbreviations: AVNRT, atrioventricular nodal reentry tachycardia; AVRT, atrioventricular reentry tachycardia; ECG, electrocardiogram; fQRS, fragmented QRS; LBBB, left bundle branch block; RBBB, right bundle branch block; VES, ventricular extrasystole.

## Data Availability

The original contributions presented in the study are included in the article/Supplementary Material, further inquiries can be directed to the corresponding author.

## Supplementary Materials

The following supporting information can be downloaded at: https://www.mdpi.com/article/10.3390/jcm13195906/s1, Table S1: Interobserver variability of the measured parameters; Table S2: Identified pathogenic and likely pathogenic mutations in cardiomyopathy-related genes in our left ventricular excessive trabeculation study population; Table S3: Identified variants of unknown significance (VUS) in cardiomyopathy-related genes in our left ventricular excessive trabeculation study population; Table S4: Correlation between the ventricular volumes and mass parameters; Table S5: The correlation of the CMR and ECG parameters in the (A) benign, (B) VUS and (C) pathogenic subgroups.
