# Supplementary material for: Electrocardiogram Features of Left Ventricular Excessive Trabeculation with Preserved Cardiac Function in Light of Cardiac Magnetic Resonance and Genetics
Source: J Clin Med. 2024 Oct 3;13(19):5906. doi: 10.3390/jcm13195906 (PMC11477278; doi:10.3390/jcm13195906)
Supplement: Supplementary file 1 [file jcm-13-05906-s001.zip › Table S5.pdf]

Table S5. The correlation of the CMR and ECG parameters in the (A) benign, (B) VUS and (C) pathogenic subgroups

A, Benign subgroup		LV_EDVi	LV_ESVi	LV_SVi	LV_EF	LV_MASSi	LV_TRABi	RV_EDVi	RV_ESVi	RV_SVi	RV_EF	RV_MASSi	RV_TRAB
QRS_duration (ms)	r	0.212	0.074	0.285	0.051	0.696*	0.264	0.401	0.188	0.448	0.188	0.275	0.212
	p	0.508	0.818	0.369	0.874	0.012	0.406	0.197	0.558	0.144	0.558	0.386	0.509
LV_SI (mm)	r	0.581*	0.586*	0.394	-0.401	0.277	0.51	0.27	0.296	0.176	-0.165	0.337	0.009
	p	0.048	0.045	0.205	0.196	0.384	0.09	0.397	0.351	0.585	0.609	0.284	0.977
RV_SI (mm)	r	0.361	0.126	0.485	0.138	0.352	0.449	0.096	0.063	0.094	-0.002	0.25	0.057
	p	0.249	0.696	0.11	0.669	0.262	0.143	0.767	0.847	0.772	0.996	0.432	0.86

B, VUS subgroup		LV_EDVi	LV_ESVi	LV_SVi	LV_EF	LV_MASSi	LV_TRABi	RV_EDVi	RV_ESVi	RV_SVi	RV_EF	RV_MASSi	RV_TRAB
QRS_duration (ms)	r	0.23	0.32	0.092	-0.32	0.582**	0.403*	0.309	0.276	0.269	-0.052	0.617**	0.582**
	p	0.258	0.111	0.656	0.111	0.002	0.041	0.124	0.172	0.184	0.799	0.001	0.002
LV_SI (mm)	r	0.268	0.198	0.245	-0.056	0.690**	0.648**	0.327	0.253	0.312	-0.025	0.584**	0.671**
	p	0.186	0.333	0.227	0.784	<0.001	<0.001	0.102	0.212	0.121	0.903	0.002	<0.001
RV_SI (mm)	r	0.293	0.301	0.202	-0.157	0.272	0.209	0.293	0.133	0.346	0.147	0.377	0.211
	p	0.146	0.135	0.322	0.444	0.179	0.306	0.146	0.518	0.083	0.473	0.058	0.302

C, Pathogenic subgroup		LV_EDVi	LV_ESVi	LV_SVi	LV_EF	LV_MASSi	LV_TRABi	RV_EDVi	RV_ESVi	RV_SVi	RV_EF	RV_MASSi	RV_TRAB
QRS_duration (ms)	r	0.522*	0.535*	0.372	-0.329	0.475	0.535*	0.763**	0.734**	0.624*	-0.193	0.592*	0.437
	p	0.046	0.04	0.172	0.231	0.074	0.04	0.001	0.002	0.013	0.49	0.02	0.103
LV_SI (mm)	r	0.421	0.262	0.479	-0.049	0.174	0.051	0.069	-0.107	0.23	0.154	0.085	0.077
	p	0.118	0.346	0.071	0.863	0.536	0.856	0.807	0.704	0.41	0.583	0.762	0.785
RV_SI (mm)	r	0.726**	0.584*	0.686**	-0.254	0.625*	0.547*	0.662**	0.641**	0.537*	-0.294	0.576*	0.443
	p	0.002	0.022	0.005	0.36	0.013	0.035	0.007	0.01	0.039	0.287	0.025	0.098

Abbreviations: * Correlation is significant at the p<0.05 level; ** Correlation is significant at the p<0.01 level; CMR: Cardiac magnetic resonance imaging; ECG: electrocardiogram; EDV: end-diastolic volume; EF: ejection fraction; ESV: end-systolic volume; i: indexed to body surface area; LV: left ventricle; QTc: corrected QT interval; r: Correlation coefficient; RV: right ventricle; SI: Sokolow-Lyon index; SV: stroke volume; TM: total muscle-mass; TPM: trabeculated and papillary muscle mass; VUS: variant of unknown significance
