# Supplementary material for: Electrocardiogram Features of Left Ventricular Excessive Trabeculation with Preserved Cardiac Function in Light of Cardiac Magnetic Resonance and Genetics
Source: J Clin Med. 2024 Oct 3;13(19):5906. doi: 10.3390/jcm13195906 (PMC11477278; doi:10.3390/jcm13195906)
Supplement: Supplementary file 1 [file jcm-13-05906-s001.zip › Table S1.pdf]

Table S1. Interobserver variability of the measured parameters

		ICC	CI lower limit	CI upper limit
CMR parameters	**LV_EDVi (ml/m2)**	0.97	0.86	0.99
	**LV_ESVi (ml/m2)**	0.97	0.89	0.99
	**LV_SVi (ml/m2)**	0.94	0.77	0.99
	**LV_EF (%)**	0.9	0.58	0.97
	**LV_TMi (g/m2)**	0.99	0.97	0.998
	**LV_TPMi (g/m2)**	0.98	0.91	0.995
	**RV_EDVi (ml/m2)**	0.99	0.97	0.998
	**RV_ESVi (ml/m2)**	0.96	0.82	0.99
	**RV_SVi (ml/m2)**	0.995	0.98	0.999
	**RV_EF (%)**	0.94	0.77	0.99
	**RV_TMi (g/m2)**	0.99	0.95	0.997
	**RV_TPMi (g/m2)**	0.99	0.95	0.997
ECG parameters	**P_duration (ms)**	0.89	0.77	0.94
	**P_amplitude (mV)**	0.79	0.09	0.928
	**PQ_duration (ms)**	0.96	0.916	0.98
	**QRS_duration (ms)**	0.97	0.877	0.991
	**LV_SI (mm)**	0.97	0.946	0.986
	**RV_SI (mm)**	0.95	0.876	0.975
	**QT (ms)**	0.86	0.729	0.932
	**QTc (ms)**	0.87	0.745	0.937
	**T_duration (ms)**	0.77	0.543	0.885
	**T_amplitude (mV)**	0.94	0.871	0.972

ICC ≥ 0.75 Excellent agreement; 0.75 > ICC > 0.4 Fair to good agreement; ICC ≤ 0.4 Poor agreement

Abbreviations: CMR: Cardaiac magnetic resonance imaging; ECG: electrogardiogram; EDV: end diastolic volume; EF: ejection fraction; ESV: end systolic volume; i: indexed to body surface area; ICC: intraclass correlation coefficient; LV: left ventricle; LVET: left ventricular excessive trabeculation; QTc: corrected QT interval; RV: right ventricle; SI: Sokolow-Lyon index; SV:
