# Supplementary material for: Electrocardiogram Features of Left Ventricular Excessive Trabeculation with Preserved Cardiac Function in Light of Cardiac Magnetic Resonance and Genetics
Source: J Clin Med. 2024 Oct 3;13(19):5906. doi: 10.3390/jcm13195906 (PMC11477278; doi:10.3390/jcm13195906)
Supplement: Supplementary file 1 [file jcm-13-05906-s001.zip › Table S2.pdf]

Table S2. Identified pathogenic and likely pathogenic mutations in cardiomyopathy-related genes in our left ventricular excessive trabeculation study population

	Gene symbol	Affected protein	variant_ID	Transcript ID	HGVSc	HGVSp
LVNC–related mutations	**TTN**	Titin	chr2-179659682-G-C	ENST00000589042	c.1212C>G	p.Tyr404Ter
			chr2-179536824-C-A	ENST00000589042	c.35101G>T	p.Glu11701Ter
			chr2-179427059-AG-A	ENST00000589042	c.83799del	p.Phe27934SerfsTer3
			chr2-179392275-G-A	ENST00000589042	c.107578C>T	p.Gln35860Ter
			chr2-179397981-TC-T	ENST00000589042	c.103360del	p.Glu34454AsnfsTer3
			chr2-179604901-TG-T	ENST00000589042	c.13058del	p.Pro4353GlnfsTer14
	**MYH7**	Myosin Heavy Chain 7	chr14-23901869-C-T	ENST00000355349	c.481G>A	p.Ala161Thr
			chr14-23897873-T-C	ENST00000355349	c.1414A>G	p.Ser472Gly
	**MYH7**	Myosin Heavy Chain 7	chr14-23885311-C-T	ENST00000355349	c.4855G>A	p.Glu1619Lys
	**TNNT2**	Troponin T2, Cardiac Type	chr1-201333463-CG-C	ENST00000509001	c.421del	p.Arg141GlyfsTer41
	**MYBPC3**	Myosin Binding Protein C3	chr11-47353740-G-A	ENST00000545968	c.3697C>T	p.Gln1233Ter
	**MIB1**	MIB E3 Ubiquitin Protein Ligase 1	chr18-19426998-C-T	ENST00000261537	c.2305C>T	p.Arg769Ter
	**LMNA**	Lamin A/C	chr1-156085004-C-A	ENST00000368300	c.295C>A	p.Arg99Ser
Other CMP-related mutations	**SCN5A**	Sodium Voltage-Gated Channel Alpha Subunit 5	chr3-38597188-G-C	ENST00000413689	c.4501C>G	p.Leu1501Val
	**KCNQ1**	Potassium Voltage-Gated Channel Subfamily Q Member 1	chr11-2799221-G-A	ENST00000155840	c.1748G>A	p.Arg583His
