# Supplementary material for: Electrocardiogram Features of Left Ventricular Excessive Trabeculation with Preserved Cardiac Function in Light of Cardiac Magnetic Resonance and Genetics
Source: J Clin Med. 2024 Oct 3;13(19):5906. doi: 10.3390/jcm13195906 (PMC11477278; doi:10.3390/jcm13195906)
Supplement: Supplementary file 1 [file jcm-13-05906-s001.zip › Table S3.pdf]

Table S3. Identified variants of unknown significance (VUS) in cardiomyopathy-related genes in our left ventricular excessive trabeculation study population

	Gene symbol	Affected protein	variant_ID	Transcript ID	HGVSc	HGVSp
LVNC–related mutations	TTN	Titin	chr2-179440214-C-T	ENST00000589042	c.70645G>A	p.Val23549Ile
			chr2-179396214-C-T	ENST00000589042	c.105128G>A	p.Arg35043His
			chr2-179497044-C-T	ENST00000589042	c.43577G>A	p.Arg14526Gln
			chr2-179664608-T-C	ENST00000589042	c.613A>G	p.Lys205Glu
			chr2-179590352-A-T	ENST00000589042	c.20579T>A	p.Leu6860Gln
			chr2-179395825-CAGA-C	ENST00000589042	c.105514_105516del	p.Ser35172del
			chr2-179434420-C-T	ENST00000589042	c.76439G>A	p.Arg25480His
			chr2-179441038-C-T	ENST00000589042	c.69821G>A	p.Gly23274Asp
			chr2-179480082-C-T	ENST00000589042	c.48590G>A	p.Arg16197His
			chr2-179485269-G-A	ENST00000589042	c.45979C>T	p.Arg15327Cys
			chr2-179454443-C-A	ENST00000589042	c.62009G>T	p.Gly20670Val
			chr2-179606268-A-C	ENST00000589042	c.11692T>G	p.Tyr3898Asp
			chr2-179411491-C-T	ENST00000589042	c.94664G>A	p.Arg31555His
			chr2-179470215-C-T	ENST00000589042	c.53807G>A	p.Arg17936His
			chr2-179593289-T-A	ENST00000589042	c.19364A>T	p.Tyr6455Phe
			chr2-179395924-C-T	ENST00000356239	c.8245A>G	p.Ile2749Val
			chr2-179401778-A-G	ENST00000589042	c.100058T>C	p.Ile33353Thr
			chr2-179397619-G-A	ENST00000589042	c.103723C>T	p.Arg34575Cys
			chr2-179428543-T-C	ENST00000589042	c.82316A>G	p.Glu27439Gly
			chr2-179399941-C-G	ENST00000589042	c.101401G>C	p.Glu33801Gln
			chr2-179462290-C-A	ENST00000589042	c.57519G>T	p.Lys19173Asn
			chr2-179440696-C-T	ENST00000589042	c.70163G>A	p.Arg23388Gln
			chr2-179428168-G-A	ENST00000589042	c.82691C>T	p.Ala27564Val
			chr2-179473599-T-A	ENST00000589042	c.52139A>T	p.Asp17380Val
	MYH6	Myosin Heavy Chain 6	chr14-23859571-G-A	ENST00000405093	c.3427C>T	p.Arg1143Trp

	**MYH7**	Myosin Heavy Chain 7	chr14-23889090-G-T	ENST00000355349	c.3690C>A	p.Asp1230Glu
	**MYPN**	Myopalladin	chr10-69948821-C-T	ENST00000358913	c.2863C>T	p.Arg955Trp
			chr10-69881632-C-G	ENST00000358913	c.437C>G	p.Ser146Cys
	**ACTC1**	Actin Alpha Cardiac Muscle 1	chr15-35083407-C-T	ENST00000290378	c.898G>A	p.Val300Ile
	**PKP2**	Plakophilin 2	chr12-33021917-C-G	ENST00000070846	c.1114G>C	p.Ala372Pro
			chr12-33003744-A-G	ENST00000070846	c.1334T>C	p.Val445Ala
	**LDB3**	LIM Domain Binding 3	chr10-88451678-G-A	ENST00000429277	c.919G>A	p.Val307Ile
	**DSP**	Desmoplakin	chr6-7565642-T-G	ENST00000379802	c.828T>G	p.Ile276Met
	**PRDM16**	PR/SET Domain 16	chr1-3347507-GGAGGAGGACGACGATGACCT-A	ENST00000270722	c.3369_3389del	p.Asp1124_Asp1130del
			chr1-3103007-C-T	ENST00000270722	c.356C>T	p.Ala119Val
			chr1-3342777 G>A	ENST00000270722	c.3272G>A	p.Arg1091Gln
	**TBX20**	T-Box Transcription Factor 20	chr7-35288360-CTTG-C	ENST00000408931	c.471_473del	p.Asn157del
	**SEPN1**	Selenoprotein N	chr1-26138338-C-T	ENST00000361547	c.1249C>T	p.Arg417Cys
	**RYR2**	Ryanodine Receptor 2	chr1-237837399 G>A	ENST00000366574	c.8594G>A	p.Gly2865Glu
			chr1-237947183-C-G	ENST00000366574	c.12171C>G	p.His4057Gln
			chr1-237758826-T-C	ENST00000366574	c.4465T>C	p.Cys1489Arg
	**DTNA**	Dystrobrevin Alpha	chr18-32428343-C-T	ENST00000598334	c.1169C>T	p.Ser390Leu
	**RYR2**	Ryanodine Receptor 2	chr1-237947092-C-T	ENST00000366574	c.12080C>T	p.Thr4027Met
**Other CMP-related mutations**	**MYO6**	Myosin VI	chr6-76564978-G-A	ENST00000369977	c.1201G>A	p.Gly401Ser
	**MYOZ2**	Myozenin 2	chr4-120085436-A-T	ENST00000307128	c.447A>T	p.Gln149His
	**ACTN2**	Actinin Alpha 2	chr1-236883470-A-C	ENST00000366578	c.427A>C	p.Ile143Leu
	**PDLIM3**	PDZ And LIM Domain 3	chr4-186444529-T-G	ENST00000284770	c.317A>C	p.Glu106Ala
			chr4-186423579-C-T	ENST00000284770	c.964G>A	p.Asp322Asn

**NEXN**	Nexilin F-Actin Binding Protein	chr1-78383933-G-A	ENST00000334785	c.422G>A	p.Arg141His
**TRIM63**	Tripartite Motif Containing 63	chr1-26380423-C-A	ENST00000374272	c.1012G>T	p.Asp338Tyr
		chr1-26384907-C-T	ENST00000374272	c.805G>A	p.Glu269Lys
		chr1-26384907-C-T	ENST00000374272	c.805G>A	p.Glu269Lys
		chr1-26384907-C-T	ENST00000374272	c.805G>A	p.Glu269Lys
**BAG3**	BAG Cochaperone 3	chr10-121431767-C-T	ENST00000369085	c.508C>T	p.Arg170Trp
		chr10-121431767-C-T	ENST00000369085	c.508C>T	p.Arg170Trp
**ABCC9**	ATP Binding Cassette Subfamily C Member 9	chr12-22025560-T-C	ENST00000261200	c.2197A>G	p.Asn733Asp
**DSG2**	Desmoglein 2	chr18-29104840-A-G	ENST00000261590	c.1003A>G	p.Thr335Ala
**GLA**	Galactosidase Alpha	chrX-100653420-C-A	ENST00000218516	c.937G>T	p.Asp313Tyr
**TNNI3**	Troponin I3, Cardiac Type	chr19-55666150-T-C	ENST00000344887	c.331A>G	p.Arg111Gly
**TGFB3**	Transforming Growth Factor Beta 3	chr14-76427292-G-A	ENST00000238682	c.1054C>T	p.Arg352Cys
