# Supplementary material for: Electrocardiogram Features of Left Ventricular Excessive Trabeculation with Preserved Cardiac Function in Light of Cardiac Magnetic Resonance and Genetics
Source: J Clin Med. 2024 Oct 3;13(19):5906. doi: 10.3390/jcm13195906 (PMC11477278; doi:10.3390/jcm13195906)
Supplement: Supplementary file 1 [file jcm-13-05906-s001.zip › Table S4.pdf]

Table S4. Correlation between the ventricular volumes and mass parameters

LVET group		LV_MASSi	LV_TRABi	RV_MASSi	RV_TRABi
**LV_EDVi**	r	**0.648****	**0.514****	**0.659****	**0.531****
	p	<0.001	<0.001	<0.001	<0.001
**LV_ESVi**	r	**0.570****	**0.590****	**0.570****	**0.493****
	p	<0.001	<0.001	<0.001	<0.001
**LV_SVi**	r	**0.527****	**0.293****	**0.544****	**0.407****
	p	<0.001	0.016	<0.001	<0.001
**RV_EDVi**	r	**0.681****	**0.563****	**0.675****	**0.604****
	p	<0.001	<0.001	<0.001	0.001
**RV_ESVi**	r	**0.634****	**0.527****	**0.657****	**0.620****
	p	<0.001	<0.001	<0.001	<0.001
**RV_SVi**	r	**0.594****	**0.437****	**0.548****	**0.465****
	p	<0.001	<0.001	<0.001	<0.001

Abbreviations: * Correlation is significant at the p<0.05 level; ** Correlation is significant at the p<0.01 level; EDV: end-diastolic volume; ESV: end-systolic volume; i: indexed to body surface area; LV: left ventricle; LVET: left ventricular excessive trabeculation; r: Correlation coefficient; RV: right ventricle; SV: stroke volume; TM: total muscle-mass; TPM: trabeculated and papillary muscle mass
